# Structural and Physicochemical Properties of *Chlorella pyrenoidosa* Neutral/Acidic Polysaccharides and Their Differential Regulatory Effects on Gut Microbiota and Metabolites in In Vitro Fermentation Model

**DOI:** 10.3390/nu17243912

**Published:** 2025-12-14

**Authors:** Ziwei Cui, Rongrong Ma, Xiaohua Pan, Chang Liu, Jinling Zhan, Tianyi Yang, Wangyang Shen, Yaoqi Tian

**Affiliations:** 1 State Key Laboratory of Food Science and Resources, Jiangnan University, Wuxi 214122, China; 6230112011@stu.jiangnan.edu.cn (Z.C.); rrma01@jiangnan.edu.cn (R.M.); panxiaohua@jiangnan.edu.cn (X.P.); liuchang670250789@jiangnan.edu.cn (C.L.); 2 School of Food Science and Technology, Jiangnan University, Wuxi 214122, China; 3 School of Biotechnology, Jiangnan University, Wuxi 214122, China; zjinling@jiangnan.edu.cn; 4 Analysis and Testing Center, Jiangnan University, Wuxi 214122, China; tianyi.sunny@jiangnan.edu.cn; 5 School of Food Science and Engineering, Wuhan Polytechnic University, Wuhan 430023, China; whwangyangshen@126.com

**Keywords:** *Chlorella pyrenoidosa* polysaccharides, structure, fecal fermentation, gut microbiota, metabolites

## Abstract

**Background/Objectives**: *Chlorella pyrenoidosa* polysaccharides (CPPs) exhibit digestion-resistant properties, with their bioactivity largely driven by gut microbiota metabolism. However, the fermentation characteristics of CPPs within the intestinal tract remain to be fully elucidated. Elucidating the utilization and metabolic processes of CPPs with respect to the gut microbiota aids in understanding the potential mechanisms underlying the biological activity of these polysaccharides. **Methods**: This work fractionated CPPs into a neutral polysaccharide fraction (CPP-1) and an acidic polysaccharide fraction (CPP-2), followed by the characterization of their structure, physicochemical properties, and in vitro fermentation characteristics. **Results**: The results demonstrated that both CPP-1 and CPP-2 were non-starch heteropolysaccharides linked primarily by α-glycosidic bonds and lacking a triple helix structure. Both samples exhibited exceptional thermal stability, high water solubility, and low viscosity properties. CPP-2 selectively promoted *Enterocloster*, whereas CPP-1 significantly enriched *Bacteroides* and *Bifidobacterium* in gut microbiota. This differential regulation may be attributable to structural variations between the polysaccharides. Functional predictions indicated that CPP-1 enhances intestinal barrier integrity and immune homeostasis, whereas CPP-2 has anti-inflammatory activity. CPP-1 and CPP-2 interventions significantly upregulated the levels of health-promoting metabolites, including nicotinamide adenine dinucleotide, putrescine, and 3′-adenosine monophosphate. CPP-1 predominantly modulated amino acid metabolic pathways, while CPP-2 could effectively regulate purine, pyrimidine, amino acid, and butanoate metabolic pathways. **Conclusions**: This work identifies CPPs (CPP-1 and CPP-2) as novel modulators of gut homeostasis and host metabolism through microbiota–metabolite axis remodeling, supporting their prebiotic potential for functional food innovation.

## 1. Introduction

Non-starch polysaccharides have garnered considerable attention due to their structural resistance to digestion, which is hypothesized to confer prebiotic effects through the selective modulation of gut microbiota [1,2,3,4]. The biological activity of polysaccharides is associated with their digestive and metabolic characteristics in the gastrointestinal tract [3]. The structure of polysaccharides determines their utilization by the gut microbiota. Furthermore, polysaccharide composition regulates microbiota profiles and metabolite production, thereby influencing host health [5]. These indigestible polysaccharides function as carbon sources for particular gut microbiota, including *Bacteroides*, *Firmicutes*, and *Bifidobacterium*, thereby supporting the proliferation and function of these bacteria [6,7]. During microbial fermentation, indigestible polysaccharides are metabolized into bioactive metabolites that exert beneficial physiological effects [8,9]. Therefore, investigating the effects of polysaccharides on gut microbiota and metabolites can help establish the mechanistic foundation for microbiota-targeted dietary interventions and elucidate the direct/indirect pathways of polysaccharide bioactivity.

*Chlorella*, a genus of green microalgae, exhibits superior traits compared to conventional terrestrial plants, including a shorter growth cycle, no land requirement for cultivation, higher photosynthetic efficiency, strong environmental adaptability, and a balanced nutrition composition [10,11]. *Chlorella pyrenoidosa* serves as a key food ingredient due to its rapid growth rate and well-documented safety [12]. Polysaccharides are a major component of *Chlorella pyrenoidosa* [13,14] and have garnered considerable attention owing to their notable biological activities. The bioactive properties of *Chlorella pyrenoidosa* polysaccharides (CPPs) are largely mediated through their microbial metabolism in the gut. Wan et al. [15] demonstrated that CPPs enhanced antioxidant capacity in *Caenorhabditis elegans* by regulating the abundance of gut microbiota. Guo et al. [16] showed that CPPs improved host lipid metabolism by modulating gut microbiota dysbiosis in high-fat-diet (HFD)-fed mice. Additionally, Lv et al. [17] and Wan et al. [18] demonstrated that *Chlorella* polysaccharides were not degraded during in vitro digestion. These polysaccharides were metabolized by the gut microbiota, remodeling gut microbiota composition and enhancing short-chain fatty acid (SCFA) production. Importantly, during polysaccharide fermentation, gut microorganisms generate diverse bioactive metabolites beyond SCFAs, such as tryptophan derivatives, and secondary bile acids [19]. These metabolites engage in direct or indirect molecular cross-talk with host physiological pathways. Nevertheless, current research on CPPs fermentation characteristics predominantly focuses on SCFA outcomes, neglecting broader changes in gut microbial metabolism. Given that microbial metabolites critically modulate host health, systemically elucidating how CPPs regulate these metabolic networks becomes imperative.

Therefore, this work establishes a comprehensive analytical framework to delineate the structural and fermentative properties of CPPs. We isolated two different fractions of CPPs, i.e., neutral (CPP-1) and acidic (CPP-2) polysaccharides, and conducted a multi-dimensional structural analysis of CPPs (CPP-1 and CPP-2), including an evaluation of their primary structure (molecular weight distribution, monosaccharide composition, functional group), higher-order conformation (circular dichroism, Congo red experiment), and processing-related properties (solubility, thermal stability, rheological behavior). We further assessed the diverse regulatory effects of CPPs on gut microbiota and metabolites by integrating 16S rDNA sequencing and untargeted LC-MS/MS metabolomic analysis in an in vitro fermentation system. Through the functional prediction of gut microbiota and pathway enrichment analysis of differential metabolites, this work provides a theoretical foundation for CPP-mediated gut health promotion and potential metabolic regulation during fermentation, thereby supporting CPPs’ development as a functional ingredient.

## 2. Materials and Methods

### 2.1. Materials

*C. pyrenoidosa* was procured from the Freshwater Algae Culture Collection at the Institute of Hydrobiology, No. FACHB-11. *C. pyrenoidosa* was harvested by laboratory culture. The specific culture parameters were as follows: the medium was TAP; the culture temperature was 26 ± 1 °C; the light–dark ratio was 24:0; the light intensity was 7500 lux; the ventilation rate was 3 L/min; and the culture period was 7 days. Following the culture, the microalgal biomass was separated by means of a centrifuge and then washed with deionized water on three occasions. Thereafter, the microalgal biomass was subjected to freeze-drying in order to obtain *C. pyrenoidosa* powder. Diethyaminoethyl (DEAE) cellulose-52 and bile salt were procured from Shanghai Yuanye Biotechnology Co., Ltd. (Shanghai, China). The remaining chemicals utilized in the present work were analytical-grade.

### 2.2. Extraction and Purification of CPP

*C. pyrenoidosa* powder was homogenized with deionized water (1:10, *w*/*v*) and extracted in a water bath at 80 °C for 3 h. The crude extract was obtained by centrifugation and then concentrated to 1/4 of its original capacity. Subsequently, 4 volumes of anhydrous ethanol were added to the extract. After being incubated overnight at 4 °C, the precipitate was collected and washed three times with anhydrous ethanol before being redissolved in water. Trypsin (5%) was added to hydrolyze excessive protein in precipitated polysaccharides for 3 h at 37 °C and pH 7.8, and then the enzyme was inactivated at 100 °C for 10 min. After removing residual proteins via the Sevag method, the supernatant was mixed with 4 volumes of anhydrous ethanol to precipitate polysaccharides. The precipitate was redissolved in water and dialyzed for 48 h to yield crude polysaccharides, which were then purified using a DEAE-52 column. The elution fractions obtained using pure water, 0.2 M NaCl, and 0.4 M NaCl were collected separately and then freeze-dried to yield CPP-1, CPP-2, and CPP-3, respectively. The presence of starch in the polysaccharide fractions was determined using an I_2_-KI solution.

### 2.3. Determination of Polysaccharide Compositions

The total sugar content was determined by the phenol–sulfuric acid method [20]. Uronic acid content was determined by the *m*-hydroxybiphenyl method [21]. Protein content was determined by the Bradford method [22].

### 2.4. Structure Characterization

#### 2.4.1. Ultraviolet (UV) Analysis

CPP-1 and CPP-2 were prepared as 0.4 mg/mL solutions, which were scanned in the wavelength range of 200–350 nm using a TU-1900 double-beam ultraviolet–visible (UV-Vis) spectrophotometer (Beijing Purkinje GENERAL Instrument Co., Ltd., Beijing, China).

#### 2.4.2. Molecular Weight (Mw) Measurements

CPP-1 and CPP-2 were prepared as 5 mg/mL solutions. The absolute molecular mass of samples was determined by high-performance size exclusion chromatography (HPSEC). The system incorporates multi-angle laser light scattering (MALLS, DAWN HELEOS 8+, Wyatt Technology Co., Santa Barbara, CA, USA). The mobile phase consisted of 0.1 M NaNO_3_ with a flow rate of 0.5 mL/min maintained at 25 °C.

#### 2.4.3. Fourier Transform Infrared Spectrum (FT-IR) Analysis

Combined, 2 mg CPP-1 and CPP-2 were placed on a sample stage of the Nicolet iS10 spectrometer (Thermo Scientific™, Waltham, MA, USA) and scanned within the 4000–700 cm^−1^ wavelength range.

#### 2.4.4. Determination of Monosaccharide Composition

After dissolving 2 mg each of CPP-1 and CPP-2 in 1 mL of 4 M trifluoroacetic acid (TFA), the mixtures were hydrolyzed at 100 °C for 4 h in a plugged test tube, followed by TFA removal through nitrogen purging. Following dissolution in 2 mL of ultrapure water, the hydrolyzed samples were filtered using a 0.22 μm microporous filter membrane, and the resulting filtrate was introduced into an autosampler vial prior to testing. The content of various monosaccharides in the hydrolyzed samples was detected by an ion chromatograph (Dionex ICS-5000+SP-5, Thermo Scientific™, Waltham, MA, USA).

#### 2.4.5. Nuclear Magnetic Resonance (NMR) Analysis of Polysaccharides

Each component (40 mg) was dissolved in 0.5 mL D_2_O, and ^1^H and ^13^C NMR spectra were acquired using an NMR spectrometer (AVANCE NEO 600MHz, Bruker Corporation, Fällanden, Switzerland).

#### 2.4.6. Circular Dichroism (CD) Spectroscopy

The 2 mg/mL CPP-1 and CPP-2 solutions were prepared with ultrapure water. Ultrapure water was used as the blank background, and CD (Chirascan V100, Applied Photophysics, Leatherhead, UK) was used for testing at 25 °C. The scanning wavelength range was set to 185–260 nm, with a bandwidth of 1 nm.

#### 2.4.7. Congo Red Experiment

A Congo red experiment was performed using the method by Nie et al. [23] with a slight modification. The 1 mg/mL CPP-1 and CPP-2 solutions were prepared with ultrapure water and mixed with 80 μmol/L Congo red solution at a volume ratio of 1:1. Different volumes (0–1.0 mL) of the mixed solution were transferred. Each volume was adjusted to 1.0 mL with 1 M NaOH and reacted for 10 min in the dark. Absorbance spectra (400–600 nm) were measured using a UV-Vis spectrophotometer, and the wavelength of maximum absorbance (λmax) was recorded.

### 2.5. Physicochemical Properties

#### 2.5.1. Steady-State Rheological Test

The effects of concentration on the steady-state rheological properties of polysaccharides were studied using an MCR302 rotary rheometer (Anton Paar, Graz, Austria). CPP-1 and CPP-2 solutions of 1 mg/mL, 3 mg/mL, 5 mg/mL, and 10 mg/mL were prepared with ultrapure water, and rheological experiments were carried out after overnight storage at 4 °C. The change in apparent viscosity at a shear rate of 0.01–100 s^−1^ was measured at 20 °C by using a cone plate with a diameter of 25 mm.

#### 2.5.2. Water Solubility of Polysaccharides

A quantity of 50 mg of CPP-1 and CPP-2 samples was dissolved in 5 mL of water and then weighed (W_0_). Samples were permitted to remain at ambient temperature for 0.5 h, during which a vortex mixer was used to mix them for 5 s every 15 min. Samples were subsequently centrifuged at 4000 r/min for 10 min. Following supernatant removal, the tubes containing the precipitate were dried at 55 °C overnight. The dried tubes were cooled to 25 °C in a desiccator and weighed; this weight was recorded as W_1_.

Solubility(%) = (W_0_ − W_1_)/W_0_ × 100 (1)

#### 2.5.3. Thermal Characteristics 

The thermal stability of CPP-1 and CPP-2 was analyzed using a thermal analyzer (NETZSCH TG 209 F1 Libra, Selb, Germany). Following precise weighing (5 mg), samples were transferred to alumina crucibles and heated from 30 to 600 °C at a constant rate of 15 °C/min.

### 2.6. In Vitro Fermentation of CPP-1 and CPP-2

#### 2.6.1. Fermentation

The in vitro fermentation of CPP-1 and CPP-2 was conducted in accordance with previously reported methods, with partial modifications [24]. Fresh fecal samples were collected from five healthy volunteers (three females and two males; aged 20–40 years) with no history of gastrointestinal diseases, no antibiotic use within the preceding three months, and no consumption of alcohol or probiotic supplements within the past month. In an anaerobic chamber, individual fecal specimens were pooled by equal weight portions to generate a composite sample, thereby minimizing inter-individual microbiome variability. The mixed fecal material was then combined with sterile phosphate buffer (0.1 M, pH 6.8) at a 1:3 (*w*/*v*) ratio. The mixture was filtered through sterile gauze, and the filtrate was combined with the base medium at a 1:10 (*v*/*v*) ratio to prepare the fecal fermentation broth. The basic medium formulation was based on the research of Liu et al. [25]. CPP-1 and CPP-2 were added as carbon sources to 5 mL of fecal fermentation broth at a concentration of 10 mg/mL each. A polysaccharide-free basal medium was used as the blank control (CK). Three technical replicates were maintained per group. The fermentation broth was cultured in an anaerobic chamber at 37 °C for 24 h. The samples obtained at 0 and 24 h were preserved at −80 °C for later analysis.

#### 2.6.2. Gut Microbiota Analysis

DNA was extracted from the samples and quantified using Qubit (Invitrogen, Carlsbad, CA, USA). Three technical replicates were processed independently throughout all steps. The V3–V4 regions of the bacterial 16S rDNA were then amplified by PCR. Paired-end sequencing (2 × 250 bp) was conducted on an Illumina NovaSeq 6000, with sequencing services provided by LC-Bio Technology Co., Ltd. (Hangzhou, China).

#### 2.6.3. Untargeted Metabolomic Analysis

Each technical replicate aliquot was prepared by adding 100 μL sample and 400 μL extraction solution (methanol–acetonitrile = 1:1 (*v*/*v*), containing deuterated internal standards) to a tube. The mixture was vortexed and sonicated in an ice-water bath for 10 min. Following centrifugation at 12,000 rpm and 4 °C for 15 min, the supernatant was analyzed by liquid chromatography–mass spectrometry (LC-MS). Differential metabolites were subsequently analyzed with MetaboAnalyst 6.0 for pathway enrichment.

### 2.7. Statistical Analysis

Data were analyzed using ORIGIN 2025 and SPSS Statistics 26. The results are expressed as the mean with standard deviation of three replicates. For comparisons between two groups, Student’s *t*-test was used, and a one-way ANOVA followed by Tukey’s post hoc test was applied for multi-group comparisons, with statistical significance defined as *p* < 0.05.

## 3. Results and Discussion

### 3.1. Purification of CPP and Composition of Purified Polysaccharide Components

**Figure 1:**
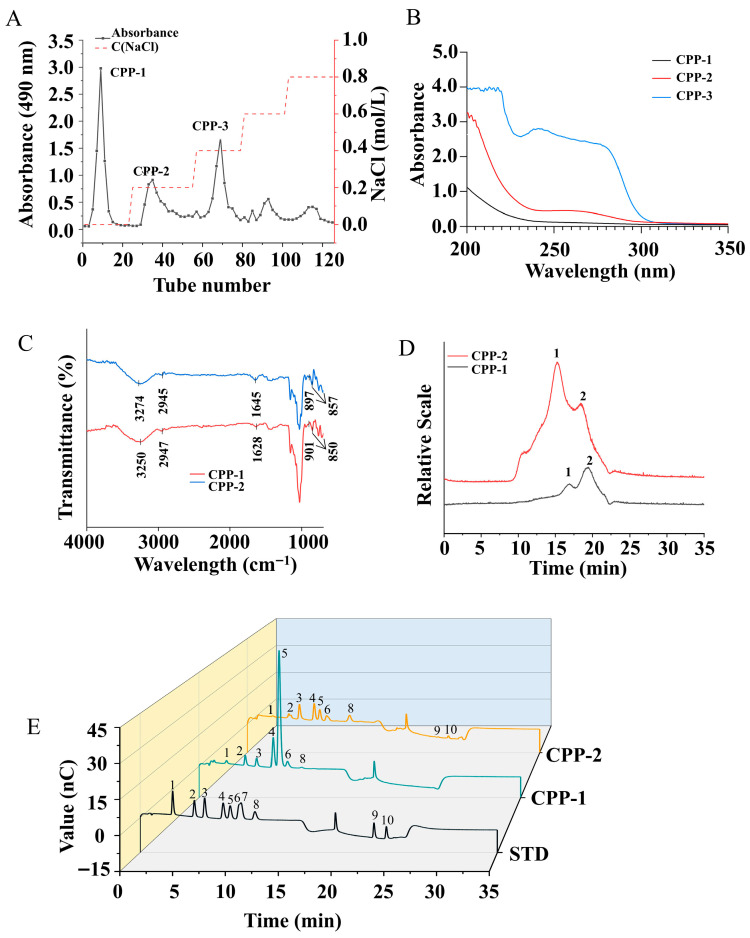
Elution curve of CPP (**A**), UV spectra (**B**) of CPP samples, FT-IR spectra (**C**), molecular weight (**D**), and monosaccharide composition (**E**) of CPP-1 and CPP-2 (peak 1: Fuc; peak 2: Rha; peak 3: Ara; peak 4: Gal; peak 5: Glc; peak 6: Xyl; peak 7: Man; peak 8: Fru; peak 9: GalA; peak 10: GlcA).

As shown in Figure 1A, CPPs were purified using a DEAE-52 column, and three purified components (CPP-1, CPP-2, and CPP-3) were obtained. No color reaction was observed upon treatment with I_2_-KI solution, confirming the absence of starch-like structures in components, thereby classifying them as non-starch polysaccharides. The UV spectra (200–350 nm) revealed that CPP-1 exhibited no absorption peaks at 260 or 280 nm, whereas CPP-2 displayed a weak absorption peak at 280 nm (Figure 1B). These results indicated that CPP-1 did not contain nucleic acids and proteins, while CPP-2 contained a small number of proteins. Both CPP-1 and CPP-2 demonstrated high polysaccharide purity and were therefore selected for further characterization. Due to its low total sugar content (≤32.2%), CPP-3 was excluded from subsequent structural and functional analyses. As shown in Table 1, the total sugar content of neutral sugar CPP-1 was remarkably higher than that of CPP-2, while the uronic acid content of acidic sugar CPP-2 was higher than that of CPP-1. These observations align with the anion-exchange separation mechanism of DEAE-52 columns, where neutral polysaccharides elute earlier than their acidic counterparts.

### 3.2. Structural Characterization

#### 3.2.1. FT-IR Analysis

The FT-IR spectra of CPP-1 and CPP-2 are shown in Figure 1C. The absorption peaks at approximately 3274 cm^−1^ and 2945 cm^−1^ were attributed to the stretching vibration of O-H and C-H [26], respectively. The absorption peak observed at 1645 cm^−1^ and the nearby region corresponded to the asymmetric stretching vibration of C=O in uronic acid [27]. The characteristic absorption peak associated with the C=O asymmetric stretching vibration of uronic acid was barely detectable in CPP-1, consistent with its absence of uronic acid. The absorption peaks in the 1200–1000 cm^−1^ range were mainly linked to C-O-C and C-O-H stretching vibrations. An absorption peak near 1030 cm^−1^ suggested the presence of a pyranose ring structure [28,29]. The presence of weak absorption peaks at 897 cm^−1^ and 857 cm^−1^ in both CPP-1 and CPP-2 suggested the presence of β- and α-glycosidic bonds within each polysaccharide [27,29].

#### 3.2.2. Mw Analysis of Polysaccharides

The Mw of polysaccharides has been demonstrated to influence their solubility, bioavailability, and biological activity. It has been shown that polysaccharides with lower Mw exhibit higher biological activity and bioavailability [30]. Generally, the Mw of polysaccharides is positively correlated with their viscosity and negatively correlated with their solubility [31]. As shown in Figure 1D and Table 1, the absolute molecular mass of samples was determined by HPSEC. Both samples exhibited two peaks. The mass fractions of the two peaks in CPP-1 were found to be 5.2% and 94.8%, respectively, and the Mw values were determined to be 1.911 × 10^5^ g/mol and 1.828 × 10^4^ g/mol, respectively. In the case of CPP-2, the mass fractions of the two peaks were 17.2% and 82.8%, respectively, and the Mw values were 8.100 × 10^5^ g/mol and 1.039 × 10^5^ g/mol, respectively. Therefore, CPP-2 possessed a larger Mw compared to CPP-1.

#### 3.2.3. Monosaccharide Composition Analysis

Both polysaccharides were identified as heteropolysaccharides based on their monosaccharide composition analysis (Figure 1E). The neutral polysaccharide CPP-1 was found to be predominantly composed of glucose (Glc), galactose (Gal), xylose (Xyl), rhamnose (Rha), and arabinose (Ara), with a molar ratio of 29.28:5.73:2.93:2.18:1.57. The Glc proportion significantly exceeded those of other monosaccharides, consistent with Chen et al.’s [32] previous findings on the monosaccharide composition of CPPs. Moreover, Glc was identified as the predominant monosaccharide composition in *C. ellipsoidea* polysaccharides [33]. No uronic acids were detected in the neutral polysaccharide CPP-1, while the acidic polysaccharide CPP-2 contained galacturonic acid (GalA) and glucuronic acid (GlcA), which corresponded to the FT-IR spectra. CPP-2 was mainly composed of Ara, Gal, fructose (Fru), and Glc. The molar ratio of Ara, Gal, Fru, Glc, Xyl, GalA, and GlcA was 3.77:3.58:3.20:2.85:1.09:0.11:0.66.

#### 3.2.4. NMR Analysis

**Figure 2:**
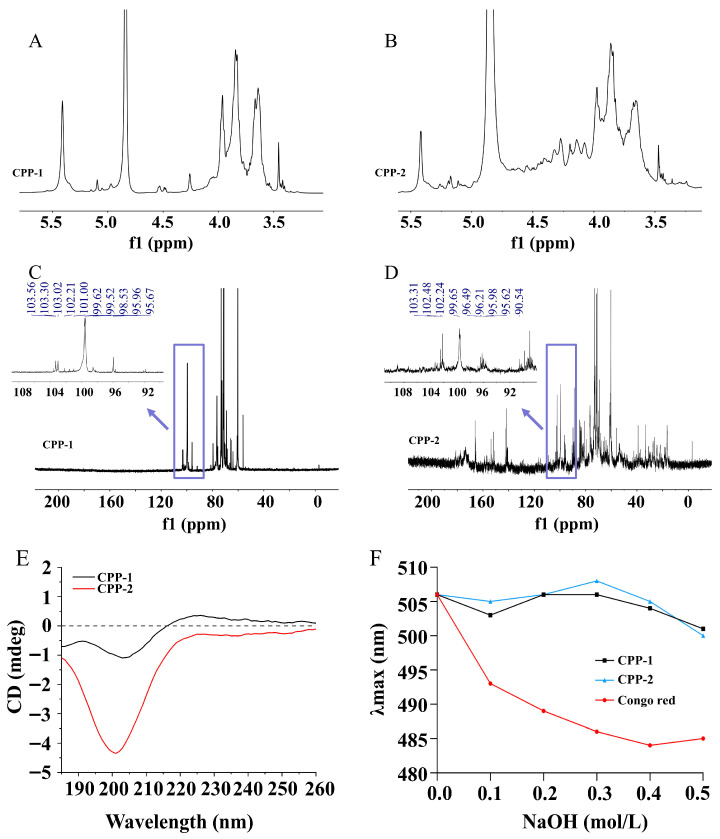
^1^H NMR spectra (**A**,**B**), ^13^C NMR spectra (**C**,**D**), CD spectra (**E**), and Congo red experiment (**F**) of CPP-1 and CPP-2.

In general, the number of sugar residues is determined based on the anomeric hydrogen (δ 4.3–5.9 ppm in the ^1^H NMR spectrum) and anomeric carbon (δ 90–112 ppm in the ^13^C NMR spectrum) of the polysaccharide. In most cases, the anomeric regions of the α-configuration appear at δ 5.1–5.8 ppm (^1^H NMR) and δ 98–103 ppm (^13^C NMR), while the corresponding anomeric regions of the β-configuration are at δ 4.3–4.8 ppm (^1^H NMR) and δ 103–106 ppm (^13^C NMR) [34]. As exhibited in Figure 2A–D, both polysaccharides had signals in the α-configuration and β-configuration regions, indicating the presence of multiple sugar residues and both α- and β-glycosidic bonds. These observations were consistent with the analysis of FT-IR. Additionally, the strong signal of CPP-1 and CPP-2 at about 5.4 ppm in the ^1^H NMR spectrum indicated that the glycosidic bonds in the two polysaccharide components were predominantly in the α-configuration [35]. The signals of CPP-1 and CPP-2 were detected at 5.35 ppm and 5.36 ppm in ^1^H NMR spectra and 99.62 and 99.65 ppm in ^13^C NMR spectra, respectively, indicating the presence of the α-anomeric configuration of glucopyranose (Glc*p*) in CPP-1 and CPP-2 [36]. The signals of 95.96 and 95.98 ppm in the ^13^C NMR spectrum corresponded to the carbon atoms of galactopyranose (Gal*p*). In the ^13^C NMR spectra, CPP-1 had no significant signal in the range of 170–210 ppm, while CPP-2 exhibited a signal at 173.55 ppm. These observations demonstrated that CPP-1 was a neutral polysaccharide, while CPP-2 contained uronic acid residues [37,38].

#### 3.2.5. Analysis of Asymmetry of Polysaccharides by CD

CD is often used to study the spatial conformation of biological macromolecules, including proteins, nucleic acids, and polysaccharides. Following the dissolution of the polysaccharide in water, the sugar chain is subject to twisting and folding, leading to an asymmetric structure and the manifestation of the Cotton effect [39]. As shown in Figure 2E, both polysaccharides exhibited negative peaks near 200 nm, indicating a negative Cotton effect. The absence of long-range ordered structures (such as helices or folds) is tentatively supported by these CD data, which suggest the possible adoption of flexible random coil or loosely curled conformations in aqueous solution.

#### 3.2.6. Analysis of Spatial Conformation of Polysaccharides by Congo Red Experiment

The spatial configuration of polysaccharides is typically a triple helix structure. Congo red has been observed to form a complex with polysaccharides exhibiting a triple helix structure, and the complex exhibits a red shift in its maximum absorption wavelength when it is exposed to alkaline conditions [40]. As demonstrated in Figure 2F, there was an evident blue shift in the maximum absorption wavelength of the blank group with increasing NaOH concentration. Conversely, both CPP-1 and CPP-2 exhibited negligible wavelength shifts, indicating no significant conformational transition across the tested alkali gradient. This absence of the characteristic red shift suggests that triple helix structures are absent in any of the polysaccharide fractions, which contrasts with the well-defined triple helix structure characterized in *Chlamydomonas reinhardtii* polysaccharides under identical alkaline conditions (Δλ > 20 nm) [41].

### 3.3. Analysis of Processing-Related Properties

#### 3.3.1. Analysis of Steady Shear Rheological Properties

**Figure 3:**
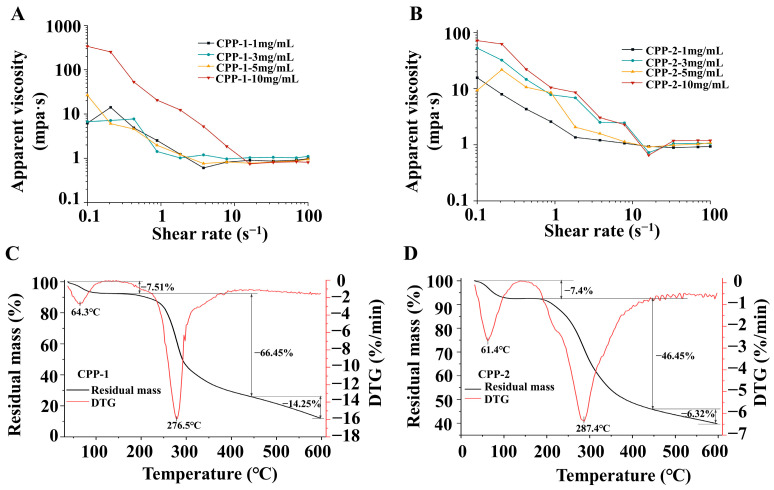
The steady-state rheological properties (**A**,**B**) and thermal stability (**C**,**D**) of CPP-1 and CPP-2.

The effect of polysaccharide concentration on the steady-state rheological properties of polysaccharides is shown in Figure 3A,B. Both CPP-1 and CPP-2 demonstrated shear-thinning behavior within the shear rate range of 0.1–100 s^−1^. Furthermore, at a concentration of 10 mg/mL, both CPP-1 and CPP-2 exhibited increased apparent viscosity and more pronounced shear-thinning behavior. The shear-thinning phenomenon can be attributed to a decline in the entanglement of polysaccharide chains, which concomitantly occurred with an increase in the shear rate. Consequently, an increase in the shear rate gives rise to an enhancement in the directional flow of the solution, which in turn leads to a reduction in the apparent viscosity of the polysaccharide [42]. Notably, this work observed low apparent viscosity in both components. Specifically, the apparent viscosity of CPP-1 solution at low concentration did not change significantly with the concentration. This phenomenon may be attributed to the relatively low Mw and an amorphous structure resulting from a loose conformation of CPP-1, which made it difficult to form effective entanglements. Despite an increase in concentration, the observed change in viscosity remained minimal.

#### 3.3.2. Analysis of Water Solubility

The solubility of polysaccharides is a critical factor influencing their application in the food industry. Generally, the water solubility of polysaccharides is associated with their Mw [31]. As demonstrated in Table 1, CPP-1 and CPP-2 exhibited excellent water solubility, with values of 99.17% and 99.12%, respectively. The excellent water-soluble characteristics of the two components may be attributed to the extraction method used and their low Mw.

#### 3.3.3. Thermal Stability Analysis

TG-DTG analysis was employed to investigate the thermal stability of polysaccharide samples. The thermogravimetric curves of the two components are shown in Figure 3C,D. The initial weight loss peaks emerged at approximately 61 °C, with weight loss rates of 7.51% and 7.4%, respectively. The primary cause of this weight loss was attributed to the evaporation of water from the polysaccharide [43]. The second-stage weight loss of CPP-1 and CPP-2 initiated at 170.0 °C and 182.1 °C, respectively. Within the temperature range of 200–450 °C, both CPP-1 and CPP-2 exhibited rapid weight loss, with mass reductions of 66.45% and 46.45%, respectively. This observation suggested that the structure of the two polysaccharide samples underwent thermal degradation within this temperature range [44]. CPP-1 and CPP-2 demonstrated relatively high thermal stability, with decomposition onset temperatures of 170.0 °C and 182.1 °C, respectively. The weight loss peaks of CPP-1 and CPP-2 appeared at 276.5 °C and 287.4 °C, respectively, which were similar to the results obtained by Noura El-Ahmady El-Naggar et al. [45]. Above 450 °C, the thermal degradation of CPP-1 and CPP-2 slowed down, with residual masses of 11.79% and 39.83% observed at 600 °C, respectively. The results indicate that both components have good thermal stability, with CPP-2 having better stability, likely due to its higher uronic acid content [45].

### 3.4. Effects of CPP-1 and CPP-2 on Gut Microbiota

**Figure 4:**
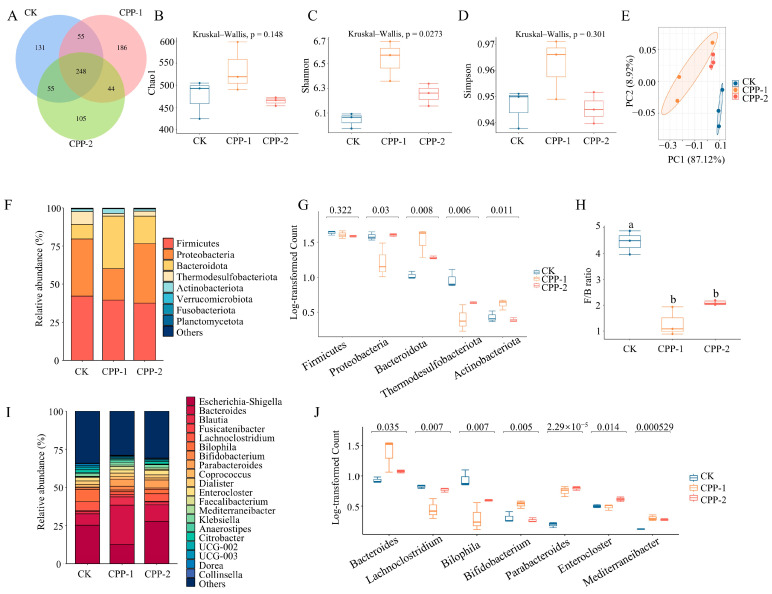
Venn diagram of shared and unique ASVs across groups (**A**), analysis of microbial Alpha diversity (**B**–**D**) and Beta diversity (**E**), composition of and difference in microbiota on phylum level (**F**,**G**) and genus level (**I**,**J**), and ratio of Firmicutes to Bacteroidota (F/B) (**H**). Different lowercase letters above box plots in panel (**H**) indicate significant differences (*p* < 0.05) among groups.

The composition of and changes in the gut microbiota influence host health. Our present study analyzed the effects of samples on the gut microbiota using 16S rDNA sequencing. As demonstrated in Figure 4A, following fermentation, the CK, CPP-1, and CPP-2 groups had 248 amplicon sequence variants (ASVs) in common. However, the CK group alone contained 131 unique ASVs, while the CPP-1 and CPP-2 groups contained 186 and 105 unique ASVs, respectively, indicating that polysaccharide intervention modified the gut microbiota composition. An evaluation of microbial α-diversity (Chao1, Shannon, and Simpson indices; Figure 4B–D) showed higher values for all indices in the CPP-1 group compared with the CK group, indicating that CPP-1 enhanced microbial richness, diversity, and evenness. The CPP-2 group exhibited an increase in the Shannon index, indicating enhanced microbial diversity. A principal coordinate analysis (PCoA; Figure 4E) of β-diversity showed separation between the CPP-1/CPP-2 groups and the CK group, indicating that both CPPs modulated gut microbiota composition.

Phylum-level gut microbiota composition differed significantly among groups (Figure 4F,G), indicating that CPP-1 and CPP-2 exerted distinct regulatory effects. Compared with the CK group, the abundance of Bacteroidota significantly increased in both the CPP-1 and CPP-2 groups, while the relative abundance of the Thermodesulfobacteriota phylum significantly decreased. This is primarily because Bacteroidota harbors numerous carbohydrate-active enzymes (CAZymes) [46], enabling the preferential utilization of polysaccharides for growth. Notably, the ratio of Firmicutes to Bacteroidota (F/B) in the sample groups (CPP-1 and CPP-2) was significantly lower than that in the CK group (Figure 4H), which was consistent with previous studies on natural polysaccharide interventions [47,48]. Li et al. [48] observed that compared to the HFD group, *Spirulina platensis* polysaccharide supplementation significantly reduced the F/B ratio. A reduced F/B ratio has been reported to be associated with obesity prevention, gut homeostasis maintenance, and improved metabolic health [49], and it is linked to alterations in the composition of gut microbiota [50]. Both *Chlorella* and *Spirulina* polysaccharides exert inhibitory effects on obesity-related inflammation in HFD-fed mice [16]. The relative abundance of the Proteobacteria phylum was reduced in the CPP-1 group, whereas that of Actinobacteriota increased markedly. The reduced Proteobacteria abundance (a phylum containing many pathogens) is associated with lower inflammation [51] and a decreased risk of gut dysbiosis [52]. These findings suggest that CPPs may improve gut and metabolic health by modulating gut microbiota composition.

To further investigate the effects of polysaccharides on gut microbiota composition, genus-level profiles post-fermentation were analyzed (Figure 4I,J). After CPP-1 and CPP-2 interventions, increases in the relative abundances of *Bacteroides*, *Parabacteroides*, and *Mediterraneibacter* were observed, while those of the opportunistic pathogens *Lachnoclostridium* and *Bilophila* decreased. Compared to the CPP-1 group, the CPP-2 group exhibited a significantly higher relative abundance of *Enterocloster*. In contrast, CPP-1 showed a marked enrichment in *Bacteroides* and *Bifidobacterium*, which may be linked to its distinct structural characteristics. Specifically, CPP-1’s lower Mw likely enhances its accessibility to gut microbiota, promoting degradation and metabolic utilization, thereby driving the dominance of *Bacteroides*. Furthermore, CPP-1’s high total sugar content (86.89 ± 2.71%) and glucose-dominant composition could account for its selective stimulation of *Bifidobacterium* proliferation. This structural dependence aligns with the study by Huang et al. [53], in which *Lentinula edodes* polysaccharides with high glucose content (Glc > 80% of monosaccharides) selectively stimulated *Bifidobacterium brevis* growth. The elevated Actinobacteriota abundance is likely related to increased *Bifidobacterium* levels. *Bacteroides* and *Parabacteroides* harbor abundant CAZymes and unique polysaccharide utilization loci (PULs) [46], enabling preferential polysaccharide utilization for growth. *Bacteroides* enhances host immunity and maintains gut microbial balance, and its increased relative abundance correlates with reduced obesity risk [54]. *Haematococcus pluvialis* polysaccharides enhance intestinal barrier integrity by restoring microbial homeostasis and promoting probiotic (e.g., *Bacteroides*) enrichment [55]. *Parabacteroides* strengthens intestinal barrier integrity and exhibits anti-obesity and anti-tumor effects [56]. Compared with the CK group, the relative abundance of *Escherichia-Shigella* decreased in the CPP-1 group, whereas that of *Blautia*, *Fusicatenibacter*, *Bifidobacterium*, and *Anaerostipes* increased. *Blautia* and *Anaerostipes* are butyrate-producing bacteria that enhance gut barrier function and suppress inflammation [57,58]. The results indicate that both samples promote the growth of beneficial bacteria, inhibit harmful bacteria, balance gut microbiota composition, and improve host health, suggesting their potential as prebiotics. Changes in gut microbiota composition suggest potential alterations in microbial metabolic functions, which were further characterized by metabolomic profiling.

**Figure 5:**
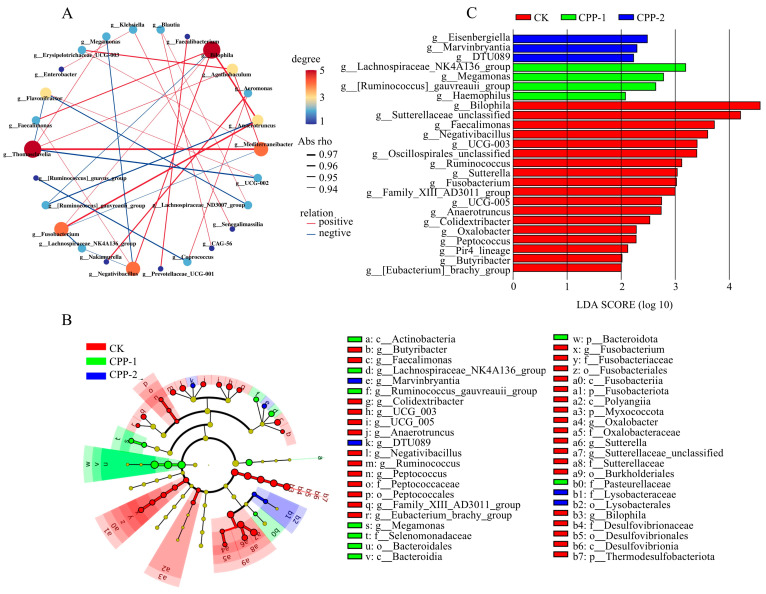
Correlation network analysis of microbial genus level (**A**), and LEfse (**B**) and LDA of microbiota (**C**).

Screening for significantly different microorganisms (*p* < 0.01) and constructing a microbial co-occurrence network (Figure 5A) identified key regulatory microbiota, revealing critical associations between *Thomasclavelia* and *Mediterraneibacter*, as well as between *Bilophila* and *Faecalimonas*, *Anaerotruncus*, or *Negativibacillus*. Differentially abundant microbial taxa between groups were determined using an LEfSe analysis combined with linear discriminant analysis (LDA > 2.0, *p* < 0.05) (Figure 5B,C). Higher LDA scores indicate a greater significance of differences. After 24 h of fermentation, beneficial bacteria were enriched in the sample groups. In the CPP-1 group, the dominant taxa included Bacteroidota (phylum), *Lachnospiraceae*_NK4A136_group, *Megamonas*, and *[Ruminococcus]_gauvreauii_group*. In the CPP-2 group, dominance was observed for *Eisenbergiella* and *Marvinbryantia*. Lachnospiraceae ferments dietary fibers to produce acetic acid and butyric acid, which serve as the primary energy sources for colonocytes [59]. *Lachnospiraceae*_NK4A136_group’s increased abundance exerts anti-inflammatory effects and maintains gut barrier integrity via butyrate-dependent mechanisms [19,60]. *Megamonas* plays a pivotal role in maintaining intestinal homeostasis through specialized carbohydrate metabolism [61]. *Eisenbergiella* modulates bile acid metabolism through interactions with bile acid monomers [62]. In summary, CPP-1 and CPP-2 interventions enriched gut health-promoting microbiota, highlighting their significant potential for promoting intestinal wellness.

### 3.5. Predicted Changes in Metabolic Pathways of Microbiota

**Figure 6:**
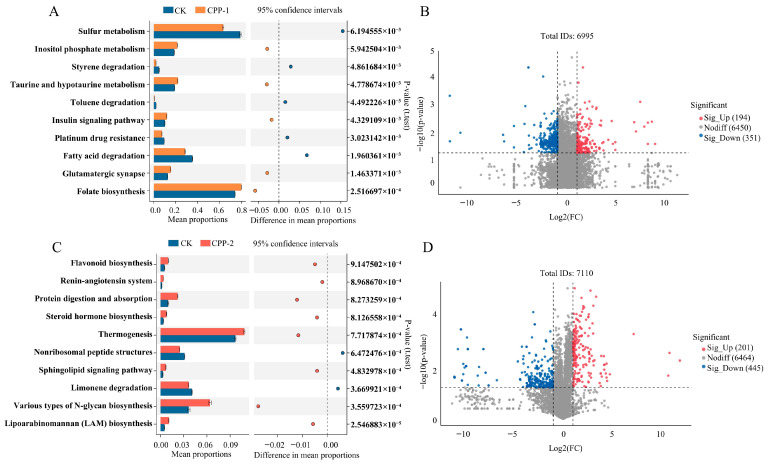
KEGG level 3 pathway enrichment between CK and CPP-1 (**A**) and CPP-2 (**C**), and volcano plots of differential KOs encoding metabolic enzymes between CK and CPP-1 (**B**) and CPP-2 (**D**).

The PICRUSt and STAMP analysis platforms, leveraging the KEGG database, were used to predict gut microbiota metabolic pathways following CPP-1 and CPP-2 interventions (Figure 6A,C). Compared to the CK group, the CPP-1 group significantly enhanced taurine and hypotaurine metabolism, insulin signaling pathways, and folate biosynthesis. The CPP-2 group significantly upregulated the biosynthesis of various types of N-glycans, protein digestion and absorption, and steroid hormone biosynthesis. Moreover, compared to CK, both CPP-treated groups exhibited significant alterations in KOs encoding metabolic enzymes (Figure 6B,D). This suggested that the gut microbiota utilized CPP-1 and CPP-2 to modulate metabolic enzyme activity. Taurine metabolism provides substrates for bile acid conjugation reactions; consequently, the elevated activity of the taurine/hypotaurine metabolic pathway increases hepatic bile acid synthesis [63]. Folate plays a vital role in maintaining immune and gut barrier homeostasis. The reduced folate levels produced by gut microbiota may trigger autoimmune diseases [64]. The activation of insulin signaling pathways enhances insulin sensitivity, whereas their inhibition induces insulin resistance [65,66]. Steroid hormone metabolites exhibit anti-inflammatory properties. Research has demonstrated that steroid hormone biosynthesis is significantly downregulated during the acute phase of COVID-19 [67]. In summary, the CPP-1 group may enhance intestinal barrier integrity, modulate immune homeostasis, and reduce diabetes risk, while the CPP-2 group demonstrates potential in suppressing pathological inflammation.

### 3.6. Effects of CPP-1 and CPP-2 on Microbial Metabolites

**Figure 7:**
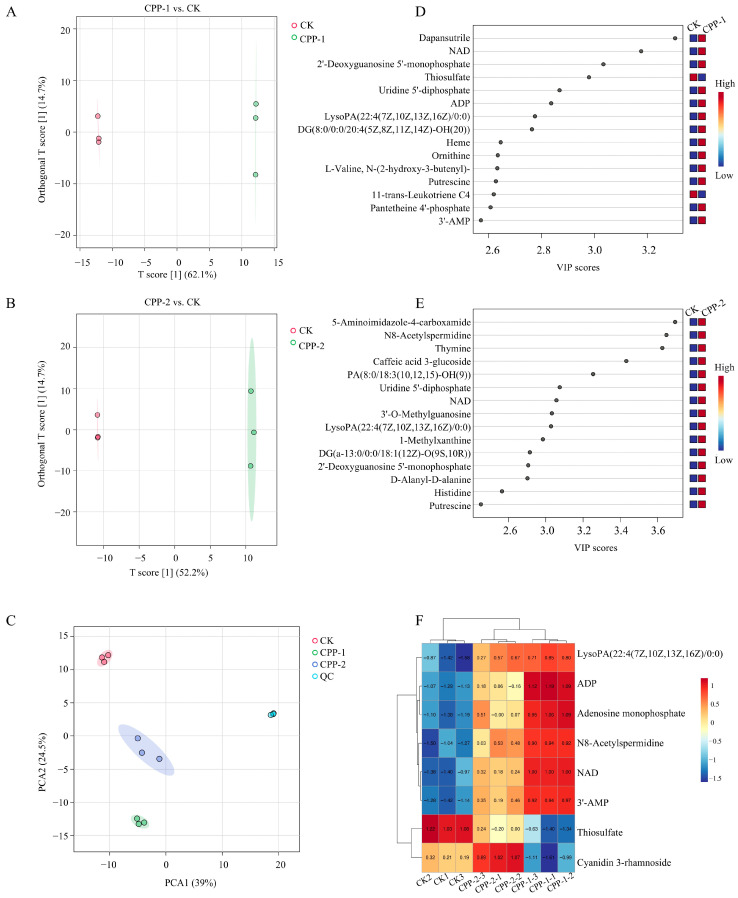
OPLS-DA score plots between CK and CPP-1 (**A**) and CPP-2 (**B**), and VIP plots between CK and CPP-1 (**D**) and CPP-2 (**E**). PCA score plots of QC, CK, CPP-1, and CPP-2 groups (**C**), and heatmap analysis of differential metabolites (**F**).

The gut microbiota exerts regulatory effects on host health through microbial metabolites. The above findings indicated that samples were able to modulate the composition of gut microbiota. To elucidate their impact on microbial metabolite profiles, untargeted metabolomics was employed to comprehensively analyze metabolite alterations. Orthogonal projections to latent structures discriminant analysis (OPLS-DA) and principal component analysis (PCA) revealed distinct separations among the CPP-1, CPP-2, and CK groups (Figure 7A–C), indicating that both interventions significantly altered the composition of gut microbial metabolites. The top 15 differentially abundant human metabolites were identified by ranking variable importance in projection (VIP) scores from OPLS-DA (Figure 7D,E). Compared to the CK group, all key metabolites in the intervention groups were upregulated, including nicotinamide adenine dinucleotide (NAD), 3′-AMP, inosinic acid (IMP), 5-aminoimidazole-4-carboxamide (AICAR), and putrescine, with the exception of thiosulfate and 11-trans-leukotriene C4, which were downregulated in the CPP-1 group. Furthermore, a heatmap analysis of eight differentially metabolites common to both sample groups compared to the CK group (Figure 7F) demonstrated that CPP-1 and CPP-2 significantly upregulated NAD and 3′-AMP compared to CK. Notably, the CPP-1 group exhibited a specific increase in adenosine monophosphate (AMP), whereas the CPP-2 group showed elevated cyanidin 3-rhamnoside levels. These metabolites have been reported to confer significant health benefits. NAD is a core regulator of cellular energy metabolism and oxidative stress responses [68]. Its upregulation enhances mitochondrial homeostasis, optimizes metabolic function, and attenuates aging processes [68,69,70]. The conversion of 3′-AMP to adenosine (and subsequently AMP) activates AMP-activated protein kinase (AMPK), modulating cellular homeostasis [71,72]. AMP enhances intestinal barrier integrity and suppresses gut inflammation through AMPK activation [73]. The AMPK-dependent upregulation of NAMPT further elevates NAD^+^ levels [74]. The metabolic effects of AICAR are potentially relevant for therapeutic interventions in type 2 diabetes mellitus, as it has been shown to effectively ameliorate metabolic dysregulation and glucose uptake [75]. Putrescine, a polyamine primarily produced by gut microorganisms, plays a pivotal function in preserving intestinal mucosal integrity and regulating metabolic pathways associated with obesity management [76,77].

**Figure 8:**
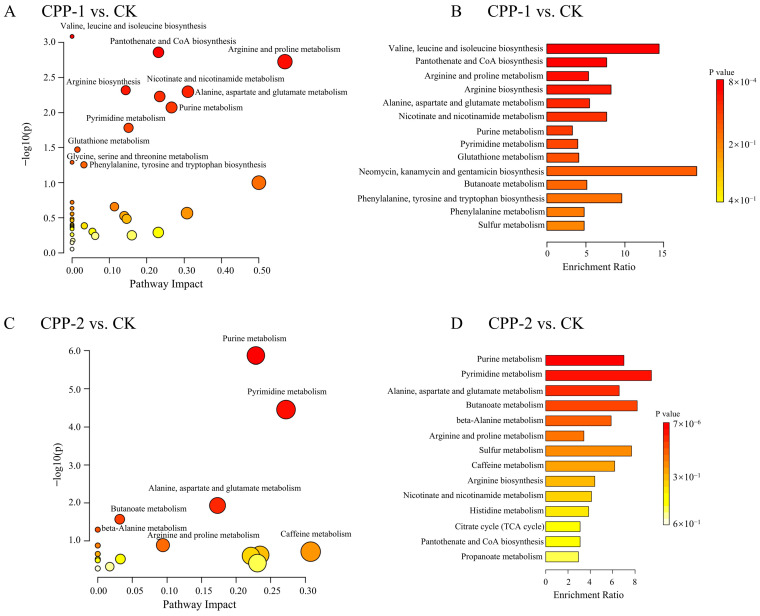
Metabolic functional analyses based on KEGG between the CK and CPP-1 groups (**A**,**B**) and between the CK and CPP-2 groups (**C**,**D**).

Metabolic pathway enrichment analysis was performed on differential metabolites between the sample and CK groups to elucidate key regulatory pathways during in vitro fermentation under CPP-1 and CPP-2 interventions (Figure 8). CPP-1 and CPP-2 interventions significantly modulated amino acid metabolism in both groups. Specifically, differentially expressed metabolites in the CPP-1 group were predominantly enriched in pathways including valine, leucine, and isoleucine biosynthesis; pantothenate and CoA biosynthesis; arginine and proline metabolism; arginine biosynthesis; alanine, aspartate, and glutamate metabolism; and purine metabolism. The CPP-2 group showed enrichment in purine, pyrimidine, alanine, aspartate, and glutamate and butanoate metabolic pathways. Among these, the upregulation of pantothenate and CoA biosynthesis correlates with enhanced antioxidant capacity and the attenuated progression of diabetic kidney disease [78,79]. Glutamate plays vital roles in cellular energy metabolism, ammonia detoxification, and brain health [80]. Dysregulated purine metabolism is linked to hyperuricemia and gout [81]. CPP-2 significantly enriched the butanoate metabolic pathway, implying enhanced butyrate biosynthesis. This aligns with prior reports that algal polysaccharides, specifically sulfated polysaccharides from *Gracilaria chouae* and fucoidan from *Undaria pinnatifida* [57,82], consistently promote butyrate-dominated SCFA profiles during in vitro fermentation. These findings suggest that the favorable biological activities of CPP-1 and CPP-2 are partially attributed to their regulation of microbial metabolites during intestinal fermentation.

### 3.7. Correlation Analysis of Gut Microbiota and Metabolites

**Figure 9:**
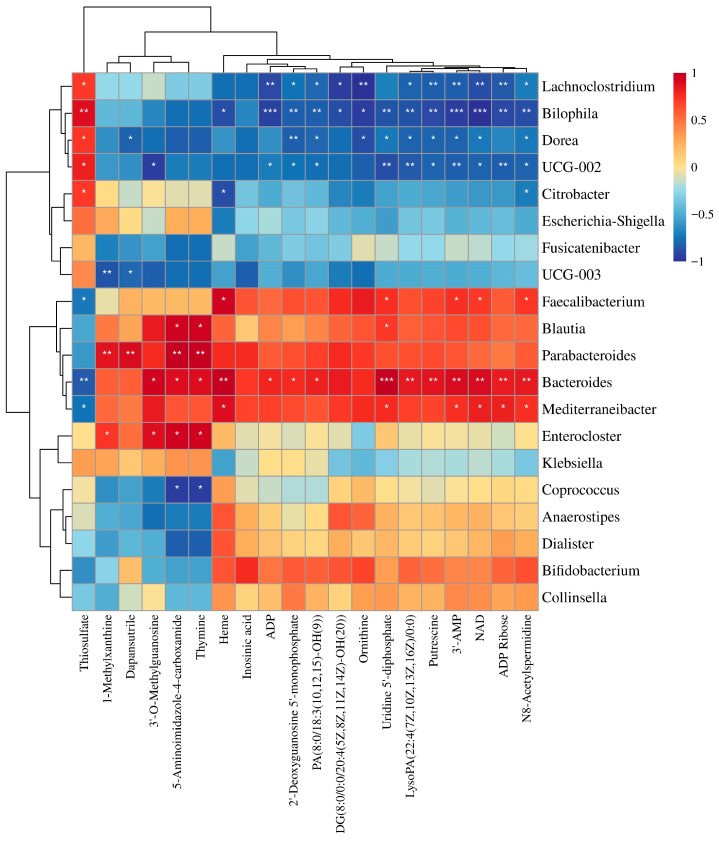
Correlation analysis between gut microbiota and metabolites. *: *p* < 0.05; **: *p* < 0.01; ***: *p* < 0.001.

A heatmap analysis of the top 20 gut microbes and metabolites was performed to further elucidate their correlations (Figure 9). Beneficial bacteria exhibited positive correlations with multiple metabolites, including energy metabolism regulators (NAD, ADP, and ADP ribose), amino acid metabolism intermediates (ornithine and putrescine), nucleic acid synthesis components (2′-deoxyguanosine 5′-monophosphate, IMP, thymine, and 3′-AMP), carbohydrate metabolism mediators (uridine 5′-diphosphate (UDP)), and lipid signaling molecules (LysoPA(22:4(7Z,10Z,13Z,16Z)/0:0), DG(8:0/0:0/20:4(5Z,8Z,11Z,14Z)-OH(20)), PA(8:0/18:3(10,12,15)-OH(9)). Among these, thymine and AICAR showed positive correlations with the abundances of *Enterocloster*, *Bacteroides*, *Parabacteroides*, and *Blautia* but negative correlations with *Coprococcus*. Furthermore, the abundance of *Bacteroides* positively correlated with heme, ADP, UDP, putrescine, 3′-AMP, and NAD, whereas *Lachnoclostridium*, *Bilophila*, and *Dorea* exhibited negative correlations with these metabolites. This work demonstrates that CPPs promote the proliferation of beneficial bacteria while inhibiting pathogenic bacteria. Therefore, CPPs may exert beneficial effects on human energy homeostasis, amino acid metabolism, carbohydrate utilization, and lipid signaling by modulating gut microbiota composition and microbial metabolite profiles.

## 4. Conclusions

Neutral (CPP-1) and acidic (CPP-2) non-starch polysaccharides, primarily linked by α-glycosidic bonds and lacking triple helix structures, were extracted from *C. pyrenoidosa*. An analysis of processing-related characteristics revealed that both components exhibited excellent thermal stability, outstanding water solubility, and low viscosity. The in vitro fermentation characteristics of CPPs were comprehensively analyzed through integrated 16S rDNA sequencing and untargeted metabolomics. The results demonstrated that both polysaccharides significantly influenced gut microbiota composition and metabolite profiles. Specifically, both components exhibited an enrichment in polysaccharide-utilizing dominant genera (*Bacteroides* and *Parabacteroides*), accompanied by reduced relative abundances of potentially pathogenic bacteria (*Lachnoclostridium* and *Bilophila*). CPP-2 selectively promoted *Enterocloster*, whereas CPP-1 significantly enriched *Bacteroides* and *Bifidobacterium* in gut microbiota. Functional predictions indicated that CPP-1 enhances intestinal barrier integrity and immune homeostasis, whereas CPP-2 has anti-inflammatory activity. Elevated levels of AMP, ADP, and heme were observed in the CPP-1 group. In contrast, the CPP-2 group had increased levels of AICAR, thymine, and cyanidin 3-rhamnoside. The CPP-1 intervention primarily modulated amino acid metabolic pathways, whereas the CPP-2 group predominantly affected purine, pyrimidine, amino acid-related, and butanoate metabolic pathways. These findings suggest that CPPs can act as promising modulators of intestinal health and host metabolism through remodeling the gut microbiota–metabolite axis. This supports their potential as prebiotic candidates that may contribute to gut homeostasis maintenance and modulate effects on energy, amino acid, and glucolipid metabolism, thereby providing a theoretical foundation for functional food innovation. However, several limitations persist in our work. Future studies should validate metabolic mechanisms through dose-escalation animal experiments assessing gut–systemic health interactions, complemented by clinical safety evaluations of *C. pyrenoidosa* polysaccharides.

## Figures and Tables

**Table 1:** Composition of and basic information about CPP-1 and CPP-2.

Sample	CPP-1		CPP-2	
Total sugar (%)	86.89 ± 2.71 ^a^		50.49 ± 1.92 ^b^	
Uronic acid (%)	ND		9.00 ± 0.11	
Protein (%)	ND		0.56 ± 0.03	
Water solubility (%)	99.17 ± 0.03 ^a^		99.12 ± 0.12 ^a^	
Molecular weight	peak 1	peak 2	peak 1	peak 2
Mass fraction (%)	5.2	94.8	17.2	82.8
Mw (g/mol)	1.911 × 10^5^	1.828 × 10^4^	8.100 × 10^5^	1.039 × 10^5^
Monosaccharide composition (molar ratio)				
Fucose	0.56		0.20	
Rhamnose	2.18		0.82	
Arabinose	1.57		3.77	
Galactose	5.73		3.58	
Glucose	29.28		2.85	
Xylose	2.93		1.09	
Fructose	0.35		3.20	
Galacturonic acid	ND		0.11	
Glucuronic acid	ND		0.66	

Note: Values are expressed as mean ± SD (*n* = 3). Different lowercase letters indicate significant differences (*p* < 0.05) among different groups. ND: Not detected.

## Data Availability

The original contributions presented in the study are included in the article, further inquiries can be directed to the corresponding author.

## References

[1] Shang Z., Ye H., Li Q., Zha X., Luo J. (2025). Potential of non-digestible polysaccharides as GLP-1 secretagogues: Action mechanisms, structure-activity relationships and clinical applications. Carbohydr. Polym..

[2] Jia R., Yang G., Lai H., Zheng Q., Xia W., Zhao M. (2024). Structural characterization and human gut microbiota fermentation in vitro of a polysaccharide from *Fucus vesiculosus*. Int. J. Biol. Macromol..

[3] Wu D., Yuan Q., Feng K., Zhang J., Gan R., Zou L., Wang S. (2022). Fecal fermentation characteristics of *Rheum tanguticum* polysaccharide and its effect on the modulation of gut microbial composition. Chin. Med..

[4] Sun Y., Yao J., Gao R., Hao J., Liu Y., Liu S. (2025). Interactions of non-starch polysaccharides with the gut microbiota and the effect of non-starch polysaccharides with different structures on the metabolism of the gut microbiota: A review. Int. J. Biol. Macromol..

[5] Yang Q., Chang S., Zhang X., Luo F., Li W., Ren J. (2024). The fate of dietary polysaccharides in the digestive tract. Trends Food Sci. Technol..

[6] Ye M., Yu J., Shi X., Zhu J., Gao X., Liu W. (2021). Polysaccharides catabolism by the human gut bacterium—*Bacteroides thetaiotaomicron*: Advances and perspectives. Crit. Rev. Food Sci. Nutr..

[7] Wu D., Yuan Q., Guo H., Fu Y., Li F., Wang S., Gan R. (2021). Dynamic changes of structural characteristics of snow chrysanthemum polysaccharides during in vitro digestion and fecal fermentation and related impacts on gut microbiota. Food Res. Int..

[8] Bai G., Xie Y., Gao X., Xiao C., Yong T., Huang L., Cai M., Liu Y., Hu H., Chen S. (2024). Selective impact of three homogenous polysaccharides with different structural characteristics from *Grifola frondosa* on human gut microbial composition and the structure-activity relationship. Int. J. Biol. Macromol..

[9] Ye K., Fu C., Du H., Chen S., Liu D., Ma G., Xiao H. (2025). Structure characterization, simulated digestion, and microbial modulation of *Pleurotus ostreatus* polysaccharides. Food Chem..

[10] Maghzian A., Aslani A., Zahedi R., Yaghoubi M. (2023). How to effectively produce value-added products from microalgae? *Renew*. Energy.

[11] Ibrahim T.N.B.T., Feisal N.A.S., Kamaludin N.H., Cheah W.Y., How V., Bhatnagar A., Ma Z., Show P.L. (2023). Biological active metabolites from microalgae for healthcare and pharmaceutical industries: A comprehensive review. Bioresour. Technol..

[12] Wu Q., Ma Y., Zhang L., Han J., Lei Y., Le Y., Huang C., Kan J., Fu C. (2023). Extraction, functionality, and applications of *Chlorella pyrenoidosa* protein/peptide. Curr. Res. Food Sci..

[13] Yuan Q., Li H., Wei Z., Lv K., Gao C., Liu Y., Zhao L. (2020). Isolation, structures and biological activities of polysaccharides from *Chlorella*: A review. Int. J. Biol. Macromol..

[14] Chang Y., Zheng F., Chen M., Liu C., Zheng L. (2024). *Chlorella pyrenoidosa* polysaccharides supplementation increases *Drosophila melanogaster* longevity at high temperature. Int. J. Biol. Macromol..

[15] Wan X.Z., Li X.Q., Liu D., Gao X., Chen Y., Chen Z., Fu C., Lin L., Liu B., Zhao C. (2021). Physicochemical characterization and antioxidant effects of green microalga *Chlorella pyrenoidosa* polysaccharide by regulation of microRNAs and gut microbiota in *Caenorhabditis elegans*. Int. J. Biol. Macromol..

[16] Guo W., Zhu S., Li S., Feng Y., Wu H., Zeng M. (2021). Microalgae polysaccharides ameliorates obesity in association with modulation of lipid metabolism and gut microbiota in high-fat-diet fed C57BL/6 mice. Int. J. Biol. Macromol..

[17] Lv K., Yuan Q., Li H., Li T., Ma H., Gao C., Zhang S., Liu Y., Zhao L. (2022). *Chlorella pyrenoidosa* Polysaccharides as a Prebiotic to Modulate Gut Microbiota: Physicochemical Properties and Fermentation Characteristics In Vitro. Foods.

[18] Wan P., Liu H., Ding M., Zhang K., Shang Z., Wang Y., Ma Y. (2023). Physicochemical characterization, digestion profile and gut microbiota regulation activity of intracellular polysaccharides from *Chlorella zofingiensis*. Int. J. Biol. Macromol..

[19] Wang J., Han L., Liu Z., Zhang W., Zhang L., Jing J., Gao A. (2023). Genus unclassified_Muribaculaceae and microbiota-derived butyrate and indole-3-propionic acid are involved in benzene-induced hematopoietic injury in mice. Chemosphere.

[20] DuBois M., Gilles K.A., Hamilton J.K., Rebers P.A., Smith F. (1956). Colorimetric Method for Determination of Sugars and Related Substances. Anal. Chem..

[21] Filisetti-Cozzi T.M.C.C., Carpita N.C. (1991). Measurement of uronic acids without interference from neutral sugars. Anal. Biochem..

[22] Bradford M.M. (1976). A rapid and sensitive method for the quantitation of microgram quantities of protein utilizing the principle of protein-dye binding. Anal. Biochem..

[23] Nie C., Zhu P., Ma S., Wang M., Hu Y. (2018). Purification, characterization and immunomodulatory activity of polysaccharides from stem lettuce. Carbohydr. Polym..

[24] Jia M., Li D., Wang R., Wang A., Strappe P., Wu Q., Shang W., Wang X., Zhuang M., Blanchard C. (2022). Gut microbiota derived structural changes of phenolic compounds from colored rice and its corresponding fermentation property. Food Funct..

[25] Liu C., Du P., Cheng Y., Guo Y., Hu B., Yao W., Zhu X., Qian H. (2021). Study on fecal fermentation characteristics of aloe polysaccharides in vitro and their predictive modeling. Carbohydr. Polym..

[26] Cai B., Zhao X., Luo L., Wan P., Chen H., Pan J. (2022). Structural characterization, and in vitro immunostimulatory and antitumor activity of an acid polysaccharide from *Spirulina platensis*. Int. J. Biol. Macromol..

[27] Zeng S., Wang B., Lv W., Li B., Xiao H., Lin R. (2024). Physicochemical properties, structure and biological activity of ginger polysaccharide: Effect of microwave infrared dual-field coupled drying. Int. J. Biol. Macromol..

[28] Zhao L., Wu L., Li L., Zhu J., Chen X., Zhang S., Li L., Yan J. (2023). Physicochemical, structural, and rheological characteristics of pectic polysaccharides from fresh passion fruit (*Passiflora edulis* f. *flavicarpa* L.) peel. Food Hydrocoll..

[29] Zhou R., Cui M., Wang Y., Zhang M., Li F., Liu K. (2020). Isolation, structure identification and anti-inflammatory activity of a polysaccharide from *Phragmites rhizoma*. Int. J. Biol. Macromol..

[30] Lee Q., Xue Z., Luo Y., Lin Y., Lai M., Xu H., Liu B., Zheng M., Lv F., Zeng F. (2024). Low molecular weight polysaccharide of *Tremella fuciformis* exhibits stronger antioxidant and immunomodulatory activities than high molecular weight polysaccharide. Int. J. Biol. Macromol..

[31] Huang F., Hong R., Zhang R., Yi Y., Dong L., Liu L., Jia X., Ma Y., Zhang M. (2019). Physicochemical and biological properties of longan pulp polysaccharides modified by *Lactobacillus fermentum* fermentation. Int. J. Biol. Macromol..

[32] Chen Y., Liu X., Wu L., Tong A., Zhao L., Liu B., Zhao C. (2018). Physicochemical characterization of polysaccharides from *Chlorella pyrenoidosa* and its anti-ageing effects in *Drosophila melanogaster*. Carbohydr. Polym..

[33] Qi J., Kim S.M. (2017). Characterization and immunomodulatory activities of polysaccharides extracted from green alga *Chlorella ellipsoidea*. Int. J. Biol. Macromol..

[34] Yao H., Wang J., Yin J., Nie S., Xie M. (2021). A review of NMR analysis in polysaccharide structure and conformation: Progress, challenge and perspective. Food Res. Int..

[35] Cao Z., Wang H., Feng T., Yao L., Sun M., Song S., Liu Q., Yu C. (2025). Physicochemical properties, techno-functional attributes, and molecular correlations of fractionated Ulva prolifera polysaccharides. LWT.

[36] Liu X., Zhu Z., Tang Y., Wang M., Wang Z., Liu A., Zhang Y. (2016). Structural properties of polysaccharides from cultivated fruit bodies and mycelium of *Cordyceps militaris*. Carbohydr. Polym..

[37] Xia L., Deji, Zhu M., Chen D., Lu Y. (2020). *Juniperus pingii* var. *wilsonii* acidic polysaccharide: Extraction, characterization and anticomplement activity. Carbohydr. Polym..

[38] Fan J., Wang Y., Yang J., Gu D., Kang S., Liu Y., Jin H., Wei F., Ma S. (2024). Anti-aging activities of neutral and acidic polysaccharides from *Polygonum multiflorum* Thunb in *Caenorhabditis elegans*. Int. J. Biol. Macromol..

[39] Sun Y., Hou S., Song S., Zhang B., Ai C., Chen X., Liu N. (2018). Impact of acidic, water and alkaline extraction on structural features, antioxidant activities of *Laminaria japonica* polysaccharides. Int. J. Biol. Macromol..

[40] Tang Z., Huang G., Huang H. (2024). Ultrasonic-assisted extraction, analysis and properties of purple mangosteen scarfskin polysaccharide and its acetylated derivative. Ultrason. Sonochem..

[41] Cui X., Guan C., Wang H., Liu Q., Zhang L., Wang Z., Zhang X. (2025). *Chlamydomonas reinhardtii* polysaccharides retard rice starch retrogradation by weakening hydrogen bond strength within starch double helices. Int. J. Biol. Macromol..

[42] Chen Y., Song L., Chen P., Liu H., Zhang X. (2023). Extraction, Rheological, and Physicochemical Properties of Water-Soluble Polysaccharides with Antioxidant Capacity from *Penthorum chinense* Pursh. Foods.

[43] Chen Y., Xue Y. (2019). Optimization of microwave assisted extraction, chemical characterization and antitumor activities of polysaccharides from *porphyra haitanensis*. Carbohydr. Polym..

[44] Liu X., Yu H., Liu Y., Qin Z., Liu H., Ma Y., Wang X. (2022). Isolation and structural characterization of cell wall polysaccharides from sesame kernel. LWT.

[45] El-Naggar N.E.-A., Hussein M.H., Shaaban-Dessuuki S.A., Dalal S.R. (2020). Production, extraction and characterization of *Chlorella vulgaris* soluble polysaccharides and their applications in AgNPs biosynthesis and biostimulation of plant growth. Sci. Rep..

[46] Qu Z., Liu H., Yang J., Zheng L., Huang J., Wang Z., Xie C., Zuo W., Xia X., Sun L. (2025). Selective utilization of medicinal polysaccharides by human gut *Bacteroides* and *Parabacteroides* species. Nat. Commun..

[47] Xu B., Feng T., Song S., Wang H., Yao L., Zhuang H., Zhang X., Liu Q., Yu C., Sun M. (2024). Effects of complex polysaccharides by *Ficus carica* Linn. polysaccharide and peach gum on the development and metabolites of human gut microbiota. Food Hydrocoll..

[48] Li T., Huang Z., Jia R., Lv X., Zhao C., Liu B. (2021). *Spirulina platensis* polysaccharides attenuate lipid and carbohydrate metabolism disorder in high-sucrose and high-fat diet-fed rats in association with intestinal microbiota. Food Res. Int..

[49] Liu N., Dai S., Fan X., Li B., Chen M., Gong P., Chen X. (2025). In vitro fermentation of *Auricularia auricula* polysaccharides and their regulation of human gut microbiota and metabolism. Int. J. Biol. Macromol..

[50] Du W., Zou Z., Ye B., Zhou Y. (2025). Gut microbiota and associated metabolites: Key players in high-fat diet-induced chronic diseases. Gut Microbes.

[51] Zhang X., Aweya J.J., Huang Z., Kang Z., Bai Z., Li K., He X., Liu Y., Chen X., Cheong K.-L. (2020). In vitro fermentation of *Gracilaria lemaneiformis* sulfated polysaccharides and its agaro-oligosaccharides by human fecal inocula and its impact on microbiota. Carbohydr. Polym..

[52] Fang Y., Zhang Q., Yu C., Xu X., Lei P., Xu H., Li S. (2024). In vitro digestion and fecal fermentation of *Tremella fuciformis* exopolysaccharides from basidiospore-derived submerged fermentation. Food Res. Int..

[53] Huang L.X., Gu F.T., Zhu Y.Y., Zhao Z.C., Li J.H., Wu J.Y. (2024). Bifidogenic properties of polysaccharides isolated from mushroom *Lentinula edodes* and enhanced immunostimulatory activities through *Bifidobacterial* fermentation. Food Biosci..

[54] Cheng J., Hu J., Geng F., Nie S. (2022). *Bacteroides* utilization for dietary polysaccharides and their beneficial effects on gut health. Food Sci. Hum. Wellness.

[55] Liu L., Zhao Z., Liu H., Xia X., Ai C., Song S., Yan C. (2024). *Haematococcus pluvialis* polysaccharides improve microbiota-driven gut epithelial and vascular barrier and prevent alcoholic steatohepatitis development. Int. J. Biol. Macromol..

[56] Liu J., Qiu H., Zhao J., Shao N., Chen C., He Z., Zhao X., Zhao J., Zhou Y., Xu L. (2025). *Parabacteroides* as a promising target for disease intervention: Current stage and pending issues. npj Biofilms Microbiomes.

[57] Li X., Peng B., Chi-Keung Cheung P., Wang J., Zheng X., You L. (2022). Depolymerized non-digestible sulfated algal polysaccharides produced by hydrothermal treatment with enhanced bacterial fermentation characteristics. Food Hydrocoll..

[58] Zeng Z., Huang H., Wang L., Lin Y., Wang B., Zheng B., Zhang Y., Pan L. (2024). Steam-exploded *Dictyophora indusiata* polysaccharide regulated gut microbiota based on dynamic in vitro stomach-intestine digestion system. Food Biosci..

[59] Jiang C., Li H., Li J., Zou G., Li C., Fang Z., Hu B., Wu W., Li X., Zeng Z. (2024). In vitro simulated digestion and fermentation behaviors of polysaccharides from *Pleurotus cornucopiae* and their impact on the gut microbiota. Food Funct..

[60] Zhou J., Li M., Chen Q., Li X., Chen L., Dong Z., Zhu W., Yang Y., Liu Z., Chen Q. (2022). Programmable probiotics modulate inflammation and gut microbiota for inflammatory bowel disease treatment after effective oral delivery. Nat. Commun..

[61] Yin Z., Zhu L., Gao M., Yu D., Zhang Z., Zhu L., Zhan X. (2024). Effects of In Vitro Fermentation of Polysialic Acid and Sialic Acid on Gut Microbial Community Composition and Metabolites in Healthy Humans. Foods.

[62] Sun J., Fan J., Li T., Yan X., Jiang Y. (2022). Nuciferine Protects Against High-Fat Diet-Induced Hepatic Steatosis via Modulation of Gut Microbiota and Bile Acid Metabolism in Rats. J. Agric. Food. Chem..

[63] Sun R., Fei F., Jin D., Yang H., Xu Z., Cao B., Li J. (2024). The integrated analysis of gut microbiota and metabolome revealed steroid hormone biosynthesis is a critical pathway in liver regeneration after 2/3 partial hepatectomy. Front. Pharmacol..

[64] Gholami H., Chmiel J.A., Burton J.P., Maleki Vareki S. (2023). The Role of Microbiota-Derived Vitamins in Immune Homeostasis and Enhancing Cancer Immunotherapy. Cancers.

[65] Shen H., Fu Y., Liu F., Zhang W., Yuan Y., Yang G., Yang M., Li L. (2024). AuCePt porous hollow cascade nanozymes targeted delivery of disulfiram for alleviating hepatic insulin resistance. J. Nanobiotechnol..

[66] Meng X., Chen B., Zhang J. (2017). Intracellular Insulin and Impaired Autophagy in a Zebrafish model and a Cell Model of Type 2 diabetes. Int. J. Biol. Sci..

[67] Liu F., Yang H., Yang T., Zhang Z., Guan L., Gao L., Ma H., Zhang H., Song N., Tong Z. (2024). Dysregulated proteasome activity and steroid hormone biosynthesis are associated with mortality among patients with acute COVID-19. J. Transl. Med..

[68] Sun S., Zhang L., Li Y., Su W., Abd El-Aty A.M., Tan M. (2024). Design and preparation of NMN nanoparticles based on protein-marine polysaccharide with increased NAD+ level in D-galactose induced aging mice model. Colloids Surf. B Biointerfaces.

[69] Waddell J., Khatoon R., Kristian T. (2023). Cellular and Mitochondrial NAD Homeostasis in Health and Disease. Cells.

[70] Katsyuba E., Mottis A., Zietak M., De Franco F., van der Velpen V., Gariani K., Ryu D., Cialabrini L., Matilainen O., Liscio P. (2018). De novo NAD+ synthesis enhances mitochondrial function and improves health. Nature.

[71] Zhang Y., Strassburger K., Teleman A.A. (2025). Remote control of AMPK via extracellular adenosine controls tissue growth. Nat. Cell Biol..

[72] Jackson E.K., Ren J., Gillespie D.G. (2011). 2′,3′-cAMP, 3′-AMP, and 2′-AMP inhibit human aortic and coronary vascular smooth muscle cell proliferation via A2B receptors. Am. J. Physiol.-Heart Circ. Physiol..

[73] Sun X., Zhu M. (2017). AMP-activated protein kinase: A therapeutic target in intestinal diseases. Open Biol..

[74] Zu Y., Wu C., Li F., Yao H., Xia Y., Zhang R., Li L., Chen S., Shi Q., Xi S. (2025). The NAMPT enzyme employs a switch that directly senses AMP/ATP and regulates cellular responses to energy stress. Mol. Cell.

[75] Višnjić D., Lalić H., Dembitz V., Tomić B., Smoljo T. (2021). AICAr, a Widely Used AMPK Activator with Important AMPK-Independent Effects: A Systematic Review. Cells.

[76] Andriyas T., Sriswasdi S., Tansawat R., Uaariyapanichkul J., Chomtho S., Visuthranukul C. (2025). Inulin supplementation modulates gut microbiota derived metabolites related to brain function in children with obesity. Sci. Rep..

[77] Zhang C., Ren H., Liu M., Li X., Sun D., Li N., Ming L. (2014). Modulation of Intestinal Epithelial Cell Proliferation, Migration, and Differentiation In Vitro by Astragalus Polysaccharides. PLoS ONE.

[78] Ma T., Liu T., Xie P., Jiang S., Yi W., Dai P., Guo X. (2020). UPLC-MS-based urine nontargeted metabolic profiling identifies dysregulation of pantothenate and CoA biosynthesis pathway in diabetic kidney disease. Life Sci..

[79] Lee Q., Han X., Zheng M., Lv F., Liu B., Zeng F. (2023). Preparation of low molecular weight polysaccharides from *Tremella fuciformis* by ultrasonic-assisted H2O2-Vc method: Structural characteristics, in vivo antioxidant activity and stress resistance. Ultrason. Sonochem..

[80] Gruenbaum B.F., Merchant K.S., Zlotnik A., Boyko M. (2024). Gut Microbiome Modulation of Glutamate Dynamics: Implications for Brain Health and Neurotoxicity. Nutrients.

[81] James A., Ke H., Yao T., Wang Y. (2023). The Role of Probiotics in Purine Metabolism, Hyperuricemia and Gout: Mechanisms and Interventions. Food Rev. Int..

[82] Wimmer B.C., Dwan C., De Medts J., Duysburgh C., Rotsaert C., Marzorati M. (2025). *Undaria pinnatifida* Fucoidan Enhances Gut Microbiome, Butyrate Production, and Exerts Anti-Inflammatory Effects in an In Vitro Short-Term SHIME^®^ Coupled to a Caco-2/THP-1 Co-Culture Model. Mar. Drugs.

